# A survey of the impact of self-supervised pretraining for diagnostic tasks in medical X-ray, CT, MRI, and ultrasound

**DOI:** 10.1186/s12880-024-01253-0

**Published:** 2024-04-06

**Authors:** Blake VanBerlo, Jesse Hoey, Alexander Wong

**Affiliations:** 1 Cheriton School of Computer Science, 200 University Ave W, N2L 3G1 Waterloo, Canada; 2 Department of Systems Design Engineering, 200 University Ave W, N2L 3G1 Waterloo, Canada

**Keywords:** Self-supervised learning, Machine learning, Representation learning, Radiology, X-ray, Computed tomography, Magnetic resonance imaging, Ultrasound

## Abstract

Self-supervised pretraining has been observed to be effective at improving feature representations for transfer learning, leveraging large amounts of unlabelled data. This review summarizes recent research into its usage in X-ray, computed tomography, magnetic resonance, and ultrasound imaging, concentrating on studies that compare self-supervised pretraining to fully supervised learning for diagnostic tasks such as classification and segmentation. The most pertinent finding is that self-supervised pretraining generally improves downstream task performance compared to full supervision, most prominently when unlabelled examples greatly outnumber labelled examples. Based on the aggregate evidence, recommendations are provided for practitioners considering using self-supervised learning. Motivated by limitations identified in current research, directions and practices for future study are suggested, such as integrating clinical knowledge with theoretically justified self-supervised learning methods, evaluating on public datasets, growing the modest body of evidence for ultrasound, and characterizing the impact of self-supervised pretraining on generalization.

**Supplementary Information:**

The online version contains supplementary material available at 10.1186/s12880-024-01253-0.

## Introduction

Significant advancements in deep computer vision has resulted in a surge of interest in applications to medical imaging. Indeed, an enormous number of publications have demonstrated the capabilities of deep learning methods in approximating diagnostic functions in radiological, histopathological, dermatological, and endoscopic imaging. Deep learning has been extensively applied in recent years to diagnostic pattern recognition tasks such as classification, object detection, and segmentation in several modalities of medical imaging [1–5].

Of course, methodological advances alone are insufficient to achieve nontrivial results for deep computer vision tasks. Large labelled datasets are the major precondition for success in supervised learning problems. Fortunately, these exist some notable examples of large, open datasets for medical images that contain expert classification labels for limited sets of conditions (e.g., CheXpert [6]). Regrettably, large medical imaging datasets containing task- or pathology-specific labels for all constituent examples do not exist in abundance [7]. Moreover, medical imaging datasets tend to contain far fewer examples than the photographic image datasets driving much of the recent progress in computer vision [8]. Obstacles such as patient privacy concerns, private corporate interests, and the need for expert labelling, hamper the production and dissemination of such datasets [9, 10]. Occasionally, situations arise in which unlabelled datasets of medical images are available. Labelling a complete dataset requires established expertise, the cost of which dwarfs the cost of crowdsourcing labels. Furthermore, tasks such as semantic and instance segmentation require greater attention to detail, significantly increasing the labelling time per example.

**Fig. 1:**
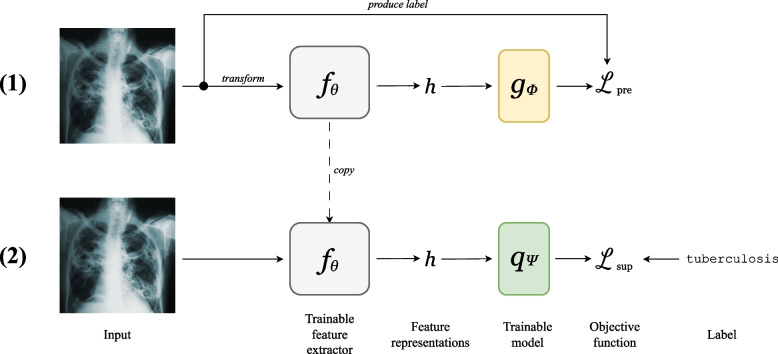
Example of a typical SSL workflow, with an application to chest X-ray classification. **(1)** *Self-supervised pretraining:* A parameterized model $g_\phi (f_\theta (\textbf{x}))$ is trained to solve a pretext task using only the chest X-rays. The labels for the pretext task are determined from the inputs themselves, and the model is trained to minimize the pretext objective $\mathcal {L}_{\text {pre}}$. At the end of this step, $f_\theta$ should output useful feature representations. **(2)** *Supervised fine-tuning:* Parameterized model $q_\psi (f_\theta (\textbf{x}))$ is trained to solve the supervised learning task of chest X-ray classification using labels specific to the classification task. Note that the previously learned $f_\theta$ is reused for this task, as it produces feature representations specific to chest X-rays

*Self-supervised learning* (SSL) has emerged as a broad strategy to learn a machine learning model that produces feature representations from unlabelled data. It is particularly beneficial when only a subset of examples in a dataset have associated labels. In brief, a machine learning model (typically a deep neural network) is trained to optimize a supervised learning objective in which the targets can be derived from the inputs themselves. In other words, a model is trained to solve a *pretext task*, which is a problem that is solvable using only the inputs and that requires no labels. *Self-supervised pretraining* refers to the optimization of a self-supervised objective to obtain a model capable of producing meaningful feature representations that capture salient information available in the inputs. The learned parameters of the pretrained model may then be used to initialize a new model that can be trained to solve a more specific supervised learning problem for which labelled data is available. Figure 1 portrays an example of the steps undertaken to pretrain a model using SSL to learn representations of chest X-rays, prior to training a multiclass chest X-ray classifier.

SSL is naturally suited to facilitate the advancement of automated diagnostic tasks with radiological images, as vast quantities of historical data are available in picture archiving and communication systems at healthcare institutions worldwide, but labels may not be present. Although accompanying radiological reports may exist in the electronic medical record, it is laborious to devise classification labels from unstructured text. Furthermore, reports may not explicitly identify all relevant negative findings for conditions of interest, opting to omit descriptions of normality. Matters are especially complicated in the context of segmentation tasks. Regardless, it is rare to encounter a fully labelled retrospectively acquired dataset. It is often necessary for experts to label at least a fraction of the dataset. Expert labelling may be prohibitively expensive in terms of monetary cost and/or time. SSL pretraining can therefore materially reduce the burden on experts to label entire radiological datasets.

The purpose of this review is to coalesce and assess evidence that the use of self-supervised pretraining can result in equivalent (and sometimes superior) performance in diagnostic tasks with small fractions of labelled radiological data. Concretely, this review offers the following:

- An overview of relevant literature that presents evidence regarding the impact of self-supervised pretraining for diagnostic tasks in radiological imaging, focusing on magnetic resonance imaging (MRI), computed tomography (CT), radiography (X-ray), and ultrasound (US).
- Identification of areas in the literature that warrant further investigation
- Recommendations for future research directions

The present work is not the first to review self-supervised approaches in medical imaging. A 2019 review by Xu [11] and a 2022 survey by Shurrab & Duwairi [12] describe common approaches to self-supervised learning and provide examples of studies that have applied it to medical imaging tasks. A 2023 systematic review by Huang et al. [13] describes the utility of SSL in medical image classification. This survey distinguishes itself from prior works in that it includes more recent literature and its scope is limited to four radiological modalitiesUnlike [13], this survey includes applications other than classification. Lastly, it addresses the theoretical underpinnings of SSL, connecting their relevance to applications in medical imaging.

The remainder of the review is organized in the following manner. First, we describe the literature search methodology that was applied to recover the studies described herein. What follows is an abridged introduction to self-supervised learning. We then present evidence for the merits of SSL as reported by a selection of recent studies – a separate section is dedicated to each of MRI, CT, X-ray, and US. Prior to the conclusion, we address gaps in the literature and summarize recommendations for future studies.

## Search methodology

The purpose of this survey is to consolidate and evaluate studies quantifying the benefit of self-supervised pretraining in the automation of diagnostic tasks concerning radiological images. A set of potentially qualifying publications as of November 2022 was found by searching the following four databases: Scopus, IEEE, ACM, and PubMed. Queries were designed to cast a wide net, including all studies whose titles, abstracts, keywords, or bodies mention medical images, CT, MRI, X-ray, ultrasound *and* self-supervised learning or contrastive learning. As will be discussed in Background section, contrastive learning is a commonly used pretext task in SSL. Appendix A gives the exact queries for each database. The search returned a total of 1226 results, which was reduced to 778 unique studies by removing duplicate and completely irrelevant papers.

Exclusion criteria were applied to the results to narrow down the body of literature to those assessing the impact of self-supervised pretraining. Studies were excluded if any of the following conditions were met:

- The study was not concerned with the radiological modalities within the scope of this survey (i.e., MRI, CT, X-ray, US).
- SSL objectives were presented in the context of semi-supervised learning, which is a family of machine learning techniques related to SSL. Like SSL, semi-supervised methods exploit unlabelled data. However, it is concerned with the simultaneous optimization of the supervised learning task of interest and an unsupervised objective.
- The study was a preprint or had not been published in a peer-reviewed periodical at the time of the literature search.
- The study applied self-supervised learning for a diagnostic task but did not compare performance on their downstream supervised learning task to a baseline (e.g., weights trained from scratch or initialized using weights pretrained on ImageNet [14]).

**Fig. 2:**
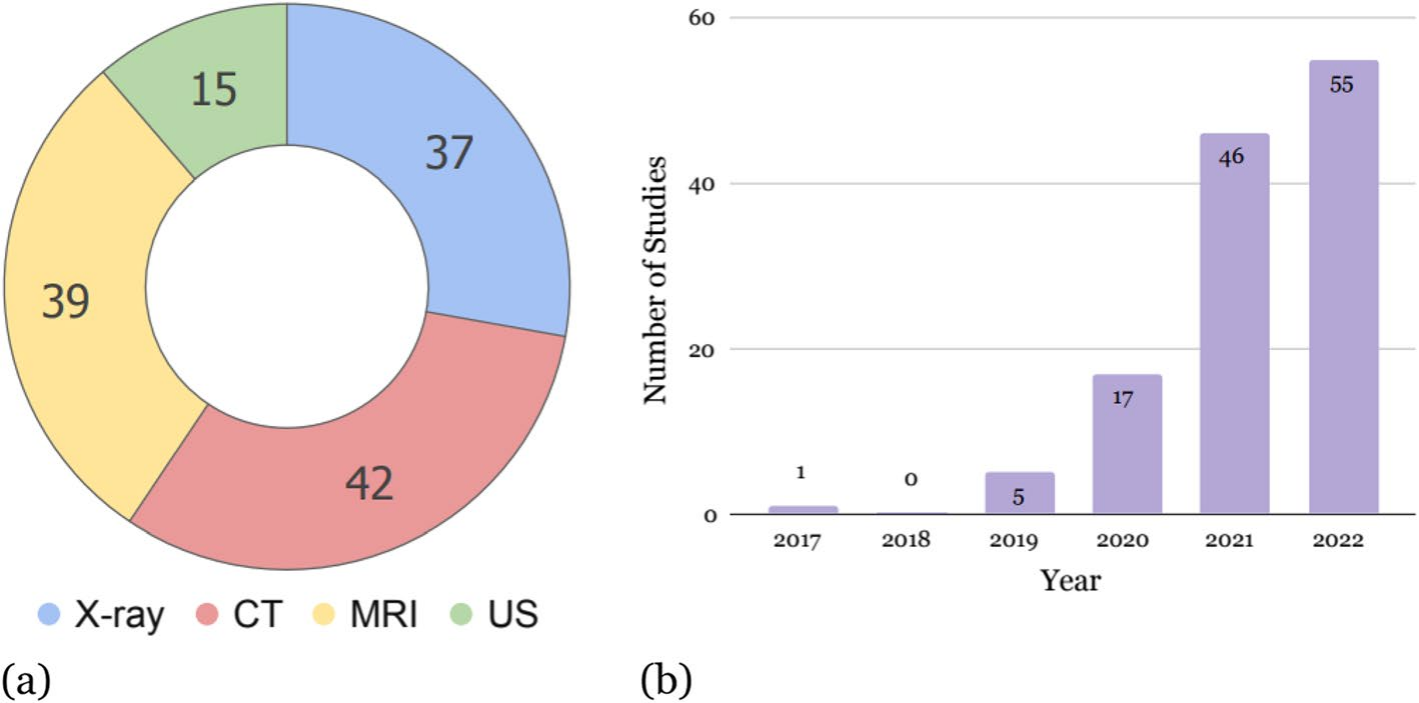
Breakdown of the papers included in this survey by **a** imaging modality and **b** year of publication

The result was a collection of 124 studies. Figure 2a visualizes the distribution of these papers by imaging modality. As shown in the figure, there are considerably less self-supervised pretraining publications geared toward ultrasound tasks than for X-ray, CT, or MRI. Figure 2b compares the number of papers in this survey published per year, reflecting the increasing interest and progress in SSL over the last couple years.

This survey directly addresses all included studies. We focused on studies that concentrate on common downstream tasks. Additionally, we attempted to highlight studies featuring replicable methods, as indicated by evaluation on public datasets and open source availability of experiment code.

## Background

### Preliminaries

In unsupervised *representation learning*, machine learning models are trained to produce compact *d*-dimensional representations of inputs that are useful for some task(s). SSL is a form of representation learning in which the objective function is formed from a pretext task whose solutions are easily obtainable from unlabelled examples. SSL distinguishes itself from supervised learning in that the objective does not depend on labels for some specific task. Like unsupervised learning, SSL aims to derive compact, low-dimensional representations for examples; however, it is distinct in that it involves optimizing supervised learning objectives.

The goal of SSL is to learn a feature extractor (also known as a *backbone* or *encoder*) that can extract high-level representations from examples. The weights of the feature extractor may then be applied to subsequent supervised learning tasks for which labels are available (often referred to as *downstream tasks*). The weights of the feature extractor may be kept stagnant or fine-tuned in the downstream learning problem. To gain intuition into the advantage of learning representations with SSL, consider the following example. Suppose a toddler is seeing different kinds of fruits for the first time. Without any feedback or external knowledge, they discover attributes of the fruits that distinguish them from others, such as colour and shape. Later, when they are taught to identify fruits by name in preschool, they apply their previously acquired knowledge about fruits to help her classify them (e.g., limes are green and round). It is likely that they have an advantage over classmates who did not eat fruit at home.

More concretely, suppose that we have a dataset of examples, $\mathcal {X}$. A *pretext task* is formulated that is solvable with knowledge of the examples. Note that the task may be defined for one or more examples. Solutions for the pretext task are taken as the labels for a self-supervised problem. An objective is defined that appropriately measures the performance of a learner at solving the pretext task.

In the context of computer vision, a backbone model, $f_\theta : \mathbb {R}^{h \times w \times c} \rightarrow \mathbb {R}^d$, is defined that maps $h \times w \times c$ images to a *d*-dimensional representation. $f_\theta$ is typically a deep neural network, parameterized by $\theta$, whose architecture embodies an inductive bias amenable to the equivariance and invariance relationships inherent to the dataset, such as a convolutional neural network (CNN). The objective is computed from the output of a secondary function $g_\phi : \mathbb {R}^{d} \rightarrow \mathbb {R}^{e}$, where $g_\phi$ is a neural network with parameter $\phi$. The pretext objective $\mathcal {L}_{\text {pre}}$ is then optimized to recover optimal weights $\theta ^*$ and $\phi ^*$.

1 $$\begin{aligned} (\theta ^*, \phi ^*) = \arg \min _{\theta , \phi } \mathcal {L}_{\text {pre}}(g_\phi (f_\theta (\textbf{x}))) \end{aligned}$$

For the chest X-ray classification example in Fig. 1, suppose chest X-ray images are passed to a CNN feature extractor $f_\theta$. The resulting feature representations $\textbf{h}$ are passed to multilayer perceptron $g_\phi$, the output of which is used to compute $\mathcal {L}_{\text {pre}}$, which quantifies performance on the pretext task.

After the objective is optimized, $g_\phi$ is customarily discarded. The backbone $f_\theta$ may then be applied for a subsequent supervised learning problem, as $f_\theta (\textbf{x})$ is a nontrivial representation of $\textbf{x}$. For a supervised learning task with examples $\mathcal {X}'$ (originating from an identical or similar distribution as $\mathcal {X}$) and corresponding labels $\mathcal {Y}$, a new model head $q_\psi : \mathbb {R}^d \rightarrow \mathbb {R}^{\text {dim}(y)}$ is initialized. $q_\psi$ receives feature representations $\textbf{h}$ as input. The model $q_\psi (f_\theta (\textbf{x}))$ is trained to minimize a loss function with respect to the labels. At this stage, $\theta$ may be held constant or fine-tuned via transfer learning. Broadly, this process is referred to as *self-supervised pretraining*. Note that it is possible that the pretrained weights $\theta$ may constitute a useful initialization for multiple downstream supervised learning problems.

### SSL approaches

The major difference between various self-supervised pretraining methods is the choice of pretext task and its optimization. Here we enumerate some broad categories of SSL methods. The intention is to provide the reader with a high-level understanding of the main approaches to SSL that may be useful when describing specific studies in the subsequent sections. These approaches are often trialled on natural images first, likely due to the high availability of benchmark datasets and broad applicability.

#### Generative methods

**Fig. 3:**
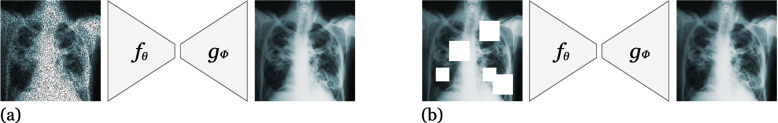
Examples of generative SSL pretext tasks

Several SSL pretext tasks are built around generating samples. The output of $g_\phi$ is an entire image or a fragment of an image. Note that generative SSL methods are not to be confused with general generative methods in machine learning (e.g. generative adversarial networks [15], denoising diffusion models [16]), where the focus is on image generation and not necessarily on producing a feature extractor. Generative SSL methods often employ an *encoder* network that learns rich feature representations. The feature representations are sent to a secondary network, frequently referred to as a *decoder*. In a self-supervised context, $g_\phi$ is the decoder and $f_\theta$ is the encoder, which is retained for downstream supervised learning. Many generative tasks are reconstructive, in that they recover a corrupted version of an image. An example of a reconstructive approach to self-supervised learning is the denoising autoencoder [17] (Fig. 3a). In the image colourization task, coloured images are generated from greyscale images, which is made possible by the availability of a dataset of coloured images [18]. Inpainting of redacted patches of images is another example of a reconstructive pretext task [19] (Fig. 3b).

#### Predictive methods

**Fig. 4:**
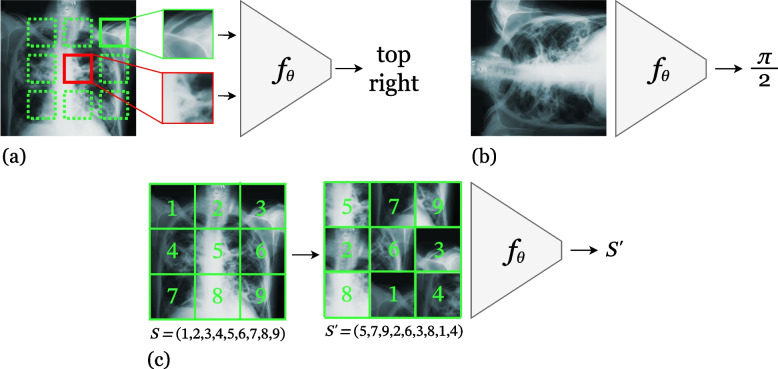
Examples of predictive SSL pretext tasks

Many custom pretext tasks have been proposed for computer vision that involve learning a specific transformation applied to images. A stochastic transformation is applied to each example, and the learner’s task is to predict or to undo the transformation. For instance, the context prediction task is defined as the problem of predicting the relative location of random image patches from unlabelled images (Fig. 4a) [20]. In the rotation prediction task, a random rotation is applied to an image and the learner must infer which rotation was applied (Fig. 4b) [21]. The jigsaw task is the unscrambling of a random permutation of all the rectangular patches in an image (Fig. 4c) [22]. Generally, the label is defined as the transformation that was applied to the image. The transformation may be stochastic in that its parameters may be sampled from some underlying distribution (e.g., the angle of a rotation being sampled from a multinoulli distribution over predefined angles).

A criticism of transformation prediction methods is that they may not be generally applicable to downstream tasks because the pretext tasks are formulated using specialized heuristics [23]. *Contrastive learning* has evolved as a generic approach for learning feature representations with fewer assumptions regarding the usefulness of particular tasks. Framed succinctly, the contrastive learning problem is to produce representations that are invariant to non-meaningful transformations. In contrastive learning, $g_\phi (\textbf{h})$ is a neural network that outputs vector *embeddings*, which need not have the same dimension as the representations $\textbf{h}$. The goal of contrastive learning is to produce embeddings $\textbf{z}_i$ that are very close (as measured by some distance function, $d(\textbf{z}_i, \textbf{z}_j)$) for *positive pairs* of examples and very far for *negative pairs*. SimCLR [23] is an example of a contrastive learning SSL method in which positive pairs are distorted versions of the same image and negative pairs are distorted versions of distinct images. The weights $\theta$ and $\phi$ are optimized such that the embeddings are close and far for positive and negative pairs respectively. To produce distorted versions of images, a series of data augmentation transformations is applied, where the parameters of the transformation are sampled from a probability distribution. Common examples of transformations include affine transformations, noise addition, and adjustments to brightness, contrast, and hue. Other notable examples of contrastive learning in SSL include MoCo [24], and PIRL [25].

**Fig. 5:**
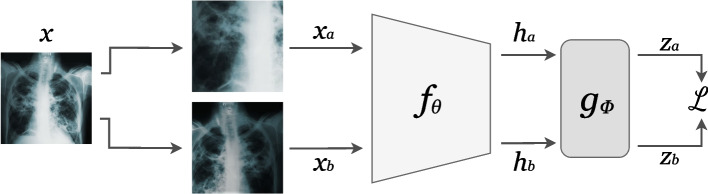
A depiction of the forward pass for a positive pair in a standard noncontrastive pretext task. An image is subject to stochastic data transformations twice, producing distorted views $\textbf{x}_a$ and $\textbf{x}_b$, which are passed through the feature extractor $f_\theta$ to yield feature representations $\textbf{h}_a$ and $\textbf{h}_b$. The projector $g_\phi$ transforms $\textbf{h}_a$ and $\textbf{h}_b$ into embeddings $\textbf{z}_a$ and $\textbf{z}_b$ respectively. Typically, the objective $\mathcal {L}$ is optimized to maximize the similarity of $\textbf{z}_a$ and $\textbf{z}_b$

A major obstacle in contemporary contrastive learning approaches is the reliance on vast quantities of negative pairs, necessitating large batch sizes [26]. Several recent publications have focused on approaches relying only on positive pairs, collectively referred to as *noncontrastive learning* (Fig. 5). Different transformations are applied to the same image to produce multiple views. $f_\theta$ and $g_\phi$ are optimized to produce embeddings that are robust to the possible views entailed by the transformation distribution, through the minimization of distance between the embeddings of positive pairs. Various strategies have been devised to avoid the problem of *information collapse*, where models learn the trivial solution of indiscriminately predicting embeddings zero vectors. Examples of methods that have reported results comparable or superior to contrastive learning include BYOL [27], Barlow Twins [26], and VICReg [28].

### Theoretical support

Until recently, SSL publications were focused primarily on introducing novel methods guided by intuitions. Some researchers have since attempted to explore the properties of SSL pretraining to better understand why they deliver such benefits and to ascertain conditions under which they will succeed.

Efforts in attempting to understanding the efficacy of optimizing performance on pretext tasks in learning downstream tasks are growing. Lee et al. [29] provided guarantees for the improved sample efficiency of pretraining with a reconstructive pretext task, in scenarios where the inputs and pretext target are conditionally independent of the labels and a latent variable. Dropping the conditional independence assumption, HaoChen et al. [30] defined a contrastive loss based on spectral decomposition and derived performance guarantees for linear classifiers trained on the feature representations from the pretraining phase. Most recently, Balestriero & LeCun [31] developed an amalgamated lens through which contemporary contrastive and noncontrastive approaches may be viewed, based on spectral analysis. They demonstrated that a selection of SSL methods (including Barlow Twins [26], VICReg [28], and SimCLR [23]) are optimal choices for solving downstream tasks as long as the relation between labels is included in the relationship between positive pairs [31]. Practitioners in all domains of computer vision are therefore encouraged to ensure that their choice of pretext task aligns appropriately with the label distribution. Those applying SSL pretraining for radiological imaging tasks should consider these results when selecting a pretext task.

## Applications In radiograph imaging

The medical imaging machine learning community has extensively reported on automatic interpretation of radiographs (X-rays). A large fraction of the effort has focused on interpretation of chest X-rays (CXR). There exists an overwhelming volume of literature describing the use of deep neural networks for CXR classification tasks. A major enabling force for this work has been the availability of large, publicly available, labelled datasets. Perhaps unsurprisingly, a flurry of studies exploring the use of self-supervised pretraining for CXR analysis followed. Despite the prevalence of open datasets, it remains difficult to directly compare publications, since pretraining and evaluation protocols differ. Here we summarize the results of such publications to understand the impact of SSL.

### Chest X-ray diagnostic tasks

Evidence has been presented in favour of self-supervised pretraining for chest X-rays, with reported benefits ranging from improved performance, label efficiency, and robustness to external data distributions. Many studies focus on the problem of identifying common respiratory conditions in CXR for which labels are available in large public datasets. A substantial fraction of publications focus on identifying COVID-19 in CXR, which is likely due to the co-occurrence of the COVID-19 pandemic and the escalation of SSL popularity.

Contrastive learning approaches have been extensively studied in the context of CXR classification. In 2020, Zhou et al. [32] introduced C2L, a joint embedding contrastive learning approach that employs a batch-wise mixup operation and a teacher network with momentum updates. Pretraining was conducted on a constellation of publicly available datasets. When fine-tuning and evaluating on Chest X-ray14 [33], CheXpert [6], and RSNA Pneumonia [34], C2L outperforms supervised models pretrained on ImageNet and self-supervised models pretrained with MoCo [24]. Other variants of MoCo have also exhibited improvement over fully supervised learning for CXR classification [35–38]. Table 1 provides average class-wise area under the receiver operating characteristic curve (AUC) reported by multiple studies for the official Chest X-ray14 test set after pretraining and training on the training set.

**Table 1:** A comparison of SSL pretraining studies that investigated chest X-ray classification using the Chest X-ray14 dataset for pretraining, training, and testing using the official splits. The table gives class-wise average test AUC as reported by the authors, when training using all available labels

Method			Initialization		
First Author [ref]	Identifier	Extractor	Random	ImageNet	SSL
Zhou [32]	C2L	ResNet-18	-	0.8150	0.8350
Zhou [32]	C2L	DenseNet-121	-	0.8290	0.8440
Ma [39]	SimMIM	ViT-B	0.7169	-	0.7955
Ma [39]	SimMIM	Swin-B	0.7704	-	0.8195
Liu [40]	S^2^MTS^2^	DenseNet-121	-	-	0.8250
Haghighi [41]	DiRA	ResNet-50	0.8031	0.8170	0.8112
Pang [42]	POPAR	Swin-B	0.7429	0.8132	0.8181

Azizi et al. [43] conducted a thorough study into the efficacy of SSL pretraining for CXR classification using a variant of SimCLR [23], reporting improvments in mean AUC of over 0.01 when pretrained on CheXpert, as compared to fully supervised models. The authors’ approach, named Multi-Instance Contrastive Learning, generalizes positive pairs to include CXRs of the same patient case, thereby exploiting information already available in the dataset to complicate the pretext task. Valuable insights were derived from their investigations. Notably, the authors found that the best-performing strategy was to initialize the weights of feature extractors with ImageNet-pretrained weights prior to conducting pretraining. Experiments also established that SSL-pretrained models outperformed fully supervised models when evaluated on Chest X-ray14, an external dataset. Other studies have reported that SimCLR pretraining (and variants) yield improvements in CXR classification [44, 45] and CXR object detection [46]. Several other publications report improvements in performance on downstream tasks using customized contrastive learning approaches for CXR diagnostic tasks [40, 47–52]

Noncontrastive approaches have also been explored for CXR diagnostic tasks. Nguyen et al. [53] applied BYOL to pretrain CXR classifiers using the ChestMNIST and PneumoniaMNIST datasets (originating from MedMNIST [54], achieving significantly higher AUC on the downstream binary classification tasks than supervised models initialized randomly or with ImageNet. Mondal et al. [55] also witnessed improvement of COVID-19 classification on the COVIDx CXR-2 dataset [56] when pretraining on CheXpert using BYOL.

Alternative pretext tasks have also yielded improvements in CXR tasks. Pang et al. [42] described the use of patch de-shuffling and recovery for pretraining vision transformers, demonstrating superior performance compared against fully supervised learning alone. Ma et al. [39] demonstrated the benefit of masked image modelling for pretraining vision transformers for various CXR tasks. Haghighi et al. [41] proposed *DiRA*, which combines discriminative methods (namely, SimSiam [57], MoCo [24], and Barlow Twins [26]), restoration of distorted images, and adversarial training into a composite pretext task. Improvements over supervised training were not observed when fine-tuning on the same dataset that was used for pretraining; however, statistically significant improvements were noted when the pretraining dataset did not match the dataset in the downstream task. Interestingly, the method outperforms each of SimSiam, MoCo, and Barlow Twins alone, indicating the possible value of composite pretext tasks. Other pretext tasks investigated for CXR classification include reconstruction of original images after transformation [58] or distortions and/or masking [59], data augmentation prediction [60], and predicting pseudo-labels generated using sample decomposition [61].

Multi-modal pretext tasks have also been explored for CXR analysis. Some public CXR datasets contain accompanying textual reports, which can be exploited to produce rich feature representations that align with physician impressions. For instance, Ji et al. [62] pretrained a network that learns similar representations for paired CXRs and reports. Müller et al. [63] demonstrate that contrastive pretraining that maximizes similarity between CXRs and reports improves performance on downstream CXR object detection and segmentation tasks on multiple public datasets, compared with image-only pretraining or fully supervised learning. In a similar approach, Tiu et al. [64] maximized the cosine similarity of paired CXR images and “Impressions” section of reports from the MIMIC-CXR dataset. In lieu of fine-tuning, the authors evaluated the trained vision transformer by providing textual prompts containing the label and taking the maximum of the logits (e.g., “pneumothorax" and “no pneumothorax") to determine the presence or absence of conditions. This zero-shot learning approach nearly matched fully supervised approaches’ performance. The success of multi-modal schemes is made possible by datasets where physician reports accompany images, such as MIMIC-CXR [65].

### Breast cancer identification

Another major diagnostic task for which many deep learning solutions have been proposed is the identification of anomalies seen on mammograms that could be cancerous. Truong et al. [66] observed that pretraining to solve the jigsaw pretext task improved prediction of malignant breast lesions when only a quarter of labels are available. You et al. [67] demonstrated that a contrastive learning pretext task outperforms the baseline. The pretext task was unique in that it considered multiple views of the same breast as positive pairs. Treating bilateral mammograms as a positive pair improved the performance of a breast cancer screening model [68]. Finally, BYOL [27] pretraining was shown to improve breast tumour segmentation [69]. In contrast with CXR studies, mammogram studies have no publicly available data, limiting the replicability of their results.

### Oral radiographs

Taleb et al. [70] investigated the utility of SimCLR, Barlow Twins, and BYOL for pretraining a CNN to detect dental caries, boosting sensitivity by up to $6\%$ and outperforming humans when fine-tuned using only 152 images. Hu et al. observed that pretraining using a reconstruction pretext task improves downstream classification and segmentation of jaw tumours and cysts.

## Applications in computed tomography

Deep computer vision has been heavily drawn upon for automated CT analysis. CT scans are volumetric scans; as a result, 3D CNNs are often leveraged. 2D CNNs are also applied for problems where a single saggital, coronal, or axial image is sufficient for the target task. Vision transformers are increasingly being studied as well. Segmentation of organs and lesions are common examples of machine learning tasks in CT. There are two major types of segmentation tasks: *semantic segmentation* consists of labelling each pixel in an image according to the class to which it belongs, and *instance segmentation* involves identifying distinct objects in an image and designating its consituent pixels. Semantic and instance segmentation tasks require greatly increased labelling time compared to classification tasks. Evidence for improved label efficiency resulting from SSL pretraining underlines its value as a cost reduction strategy. In this section, we explore the impact of SSL pretraining for CT, categorized by application.

### Lung nodule detection & segmentation

The LIDC-IDRI database is a large, labelled, public collection of CT scans with lung nodule annotations and segmentation masks [71]. It comprised the dataset for the LUNA2016 challenge [72], which was an open competition aimed at finding machine learning solutions to lung cancer screening. It became a common CT computer vision benchmark, and many SSL studies have utilized it.

Referenced by multiple succeeding publications, Models Genesis [73] devised a restorative approach for pretraining on subvolumes of 3D medical images. The approach involved applying transformations such as nonlinear translation, pixel shuffling, cropping, and masking to the subvolume. An encoder-decoder CNN is pretrained to restore the subvolumes. The encoder is reused for downstream classification tasks, while the entire pretrained encoder-decoder is used for downstream segmentation tasks. The pretrained models are available upon request, strengthening the replicability of their results. Building on Models Genesis, the Semantic Genesis [74] approach adds a classification loss to the reconstruction loss. The classification task is to predict which class a subregion belongs to, where the classes are constructed for clusters in the latent space of a pretrained autoencoder, which the authors claim contain rich semantic features. The Parts2Whole [75] pretext task involves reconstructing a CT volume from a random subvolume. Since the above three methods were tested on the same LUNDA2016 splits, they can be directly compared (see Table 2). For 3D volume inputs, the above methods are superior to training a lung nodule detector from scratch. However, 2D slice-based models pretrained with Models Genesis or Semantic Genesis do not outperform fully supervised models initialized with ImageNet-pretrained weights. Other SSL approaches that report improvement over training from scratch have been reported for this problem, but are not directly comparable due to having been trained and/or evaluated on different subsets of LIDC-IDRI [76–80]. Several of the aforementioned studies have also observed increase in performance for lung nodule segmentation on [71] when pretraining using their own or preceding SSL methods [73–75, 80].

**Table 2:** A comparison of SSL pretraining studies for 2D and 3D CNNs that investigated lung nodule detection using the LIDC-IDRI dataset and the LUNA 2016 splits. The table gives test AUC as reported by the authors

Method		Initialization		
First Author [ref]	Identifier	Random	ImageNet	SSL
Zhou [73]	Models Genesis (2D)	0.9603	0.9779	0.9745
	Models Genesis (3D)	0.9603	-	0.9834
Haghighi [74]	Semantic Genesis (2D)^a^	0.9425	0.9750	0.9750
	Semantic Genesis (3D)	0.9425	-	0.9847
Feng [75]	Parts2Whole (3D)	0.9425	-	0.9867

^a^Values were estimated via visual inspection, since results were reported in a chart

### Pulmonary embolism detection & segmentation

Constructed from the private dataset used by Tajbakhsh et al. [81], ECC is a private benchmark that contains chest CT scans, along with labels that differentiate true pulmonary emboli from false positives. Models Genesis [73] and Parts2Whole [75] both report a substantial improvement over training 3-dimensional (3D) CNNs from scratch, with Models Genesis achieving slightly higher test AUC than Parts2Whole in a direct comparison. Once again, 2D CNNs pretrained with Models Genesis outperform training from scratch, but do not clearly outperform models initialized with ImageNet-pretrained weights. Redesigning conventional discriminative pretext tasks (e.g., jigsaw, rotation) to include reconstructive and adversarial regularizers, Guo et al. [80] observe consistent improvement on ECC using all pretext tasks. It is unclear how the authors of the above studies procured the original private dataset first used in [81].

RSNA-PE is a public dataset containing pulmonary embolism labels for chest CT examinations. Islam et al. [82] pretrained various 2D CNN architectures on ImageNet using an assortment of previously proposed SSL methods, finding that downstream performance on the RSNA-PE test set improved for half of the self-supervised methods studied, compared to initialization with ImageNet-pretrained weights. Their mixed results are unsurprising, considering that they did not pretrain using CT data. Ma et al. pretrained vision transformers on RSNA-PE using SimMIM [83], a masked image modelling pretext task, observing a statistically significant improvement in test AUC.

### Cerebral hemorrhage detection

An assortment of CT classification tasks have benefitted from self-supervised pretraining. Zhuang et al. [84] trained a 3D CNN classifier to detect cerebral hemorrhage, applying a custom pretext task they playfully liken to solving a Rubik’s cube. The pretext task was to predict the random permutation and rotation applied to the 8 subvolumes of the cuboid input. Their custom pretraining resulted in $11.2\%$ higher accuracy than training from scratch. Subsequent work modified the task by randomly masking subvolumes, adding a prediction head to classify the masking pattern applied [85]. The change resulted in a $1\%$ improvement in accuracy over their previous study. However, the accuracy is lower than in the first study, raising the question of whether the same train/test partitions were applied. Further building on this work, Zhu et al. [86] form an aggregative pretext task that solves multiple proxy tasks, including their prior Rubik’s cube method. The pretext tasks are iteratively added after evaluating fine-tuning experiments, and it is unclear if the authors refer to test or training performance. They report improvements over training from scratch using all proxy tasks studied, the greatest accuracy boost being $17.22\%$.

### COVID-19 diagnosis

As in CXR applications, there exist multiple applications to COVID-19 diagnosis in CT. Early in the pandemic, Li et al. [87] extend their previous work (Rubik’s cube, introduced above in Cerebral hemorrhage detection section) by randomly masking subvolumes and predicting the mask. It is unclear how this method differs from the masking task delineated in [85] – in fact, the paragraphs describing the masking pretext task are nearly identical in [85] and [87]. The authors report an increase in precision but decrease in recall, compared to training from scratch. Interestingly, Ewen & Khan [88] achieve better performance on the public COVID-CT dataset [89] by employing a seemingly trivial pretext task of predicting whether a CT scan has been horizontally reflected across the saggital plane. Lu & Dai [90] conducted two rounds of contrastive pretraining using MoCo – one on the LUNA2016 [72] lung nodule analysis challenge dataset and a second on an expanded version of COVID-CT. When evaluating on the COVID-CT test set, they observed performance improvement compared to ImageNet pretraining. Hochberg et al. [91] pretrained a StyleGAN and used the convolutional discriminator to initialize a CNN for fine-tuning, observing an improvement over both training from scratch and pretraining with MoCo for COVID-19 detection. Focusing instead on vision transformers, Gai et al. [79] found that pretraining with DINO [92] substantially improved the AUC of a COVID-19 classifier on the public COVID-CTset [93] dataset.

Moving beyond classification, Gao et al. [94] found that pretraining using reconstruction or denoising tasks improved the Dice score of a model trained to segment COVID-19 lesions on CT images. Since their pretext tasks were generative, they were able to use the weights of both the encoder and decoder to initialize their downstream model. However, their method requires a separate diagnostic procedure, since it was trained on a private dataset consisting only of CT examinations from patients with COVID-19.

### Organ & tumour segmentation

Multiple studies report results for pancreatic tumour segmentation on a 4-fold cross validation on the public NIH Pancreas-CT dataset [95]. Custom pretext tasks for this problem include reconstruction after shuffling CT slices [96], reconstruction of scrambled subvolumes [97], and contrastive learning using inter- and intra-case pairwise relationships [98]. Table 3 compares results reported by these studies. [99] and [100] report improved segmentation of pancreatic tumours in the public MSD dataset [101], compared to training from scratch.

**Table 3:** A comparison of SSL pretraining studies for segmentation in NIH Pancreas-CT [95]. The mean Dice score on the standard 4-fold cross validation is reported

Method		Initialization	
First Author [ref]	Identifier	Random	SSL
Zheng [96]	Slice Shuffle	0.8569	0.8621
Tao [97]	Rubik’s cube++	0.8209	0.8408
Yang [98]	VoxSeP (3D)	0.8353	0.8571

The LiTS2017 dataset is a publicly available benchmark for liver tumour segmentation [102]. Multiple studies have utilized it to trial their SSL approaches, including the following aforementioned works: Models Genesis [73], Parts2Whole [75], United [80], and self-supervised StyleGAN [91]. Table 4 compares the intersection over union (IoU) reported by the first three studies – [91] formulates the LiTS2017 benchmark as a classification task and observed an improvement in AUC when pretraining with their StyleGAN-based approach. Table 4 gives strong evidence in favour of pretraining 3D CNNs for liver tumour segmentation. However, once again the 2D CNNs pretrained using Models Genesis on LUNA2016 were not superior to fully supervised 2D CNNs initialized with ImageNet-pretrained weights.

**Table 4:** A comparison of SSL pretraining studies for liver tumour segmentation using 3D CNNs on the LiTS2017 benchmark. We display the intersection over union (IoU) reported by each study

Method		Initialization	
First Author [ref]	Identifier	Random	SSL
Zhou [73]	Models Genesis (3D)	0.7976	0.8510
Feng [75]	Parts2Whole	0.7782	0.8670
Guo [80]	United	0.7782	0.8653

The BTCV benchmark contains abdominal CT scans with segmentation labels for 13 abdominal organs [103]. Tang et al. [104] pretrained vision transformers using a composite loss with reconstructive, contrastive, and rotation classification terms, following random masking and rotation of CT volumes. They observed that the combination of all regularizers was superior to a subset of them or training from scratch. Jiang et al. [105] applied masked image modelling and self distillation to train vision transformers, evaluating on BTCV. Table 5 compares SSL pretraining approaches that evaluate on the expansive BTCV benchmark. In all cases, pretraining appears to outperform training from scratch by a slim margin.

**Table 5:** A comparison of SSL pretraining studies for the BTCV benchmark. We display the average Dice score across the BTCV tasks reported by each study

Method		Initialization	
First Author [ref]	Identifier	Random	SSL
Yang [98]	VoxSeP	0.8428	0.8601
Tang [104]	Swin UNETR	0.8343	0.8472
Jiang [105]	SMIT	0.8500	0.8778

Zheng et al. [106] trialled a different composite loss for a hierarchical pretext task. They pretrained using multiple datasets, formulating classification losses for contrastive learning, task prediction, and group prediction (where a group is a subset of anatomically similar datasets), along with a reconstruction loss. They argued that the use of these regularizers would facilitate the integration of hierarchical knowledge embodied by the relationship of the datasets to one another into the feature extractor. Through ablation studies, they found that all components of the regularizer led to the best performance and that their approach was superior to a standard encoder-decoder architecture.

Lastly, multiple studies have observed improvement for self-supervised pretraining for the task of organ-at-risk segmentation, which plays a vital role in radiotherapy. Pretext tasks included multi-view momentum contrastive learning [107], predicting inter-slice distance [108], and an extension of Models Genesis with patch swapping [109]. However, the experimental validity of [107] is severely limited because the test set was included in the dataset used for pretraining.

### Other CT diagnostic tasks

A plethora of studies investigate self-supervised pretraining for a variety of diagnostic tasks on CT, demonstrating its merit. Examples of other tasks explored include kidney tumour classification (with the public KiTS19 dataset [110]) [111], liver lesion classification [112, 113], renal cell carcinoma grading [114], grading of non-alcoholic fatty liver disease [115], object detection for lesions [116] and organs [117], coronary vessel segmentation [118], whole heart segmentation (with the public WHS-CT dataset [119]) [120], abdominal muscle segmentation [121], and pneumothorax segmentation [122].

## Applications in magnetic resonance imaging

As another 3D modality, MR examinations are cumbersome to segment. Unsurprisingly, there exist several studies that have leveraged self-supervised pretraining to derive value from unlabelled MRI data. Here we enumerate and evaluate evidence regarding the effect of pretraining for diagnostic tasks with MRI.

### Brain MRI diagnostic tasks

Brain tumour segmentation is a frequently studied downstream task for which open datasets exist. The BraTS challenge [123] is a common benchmark for multi-modal MRI segmentation. It contains anatomically aligned T1, contrast T1, T2, and FLAIR brain MR scans, along with ground truth segmentation labels for brain tumours. The BraTS dataset has been updated multiple times, and is often referred to in conjunction with the year the challenge was held. Several reconstructive pretext tasks have been proposed for this problem. Chen et al. [117] adopted a reconstructive task, corrupting MRI slices by swapping locations of square patches of pixels. They observed an improvement in nearly all performance metrics when using $25\%$ and $50\%$ of the dataset. However, they did not perform a comparison using all of the available training labels. Kayal et al. [124] presented an inpainting pretext task where 3D supervoxels were redacted from the volume. Their 3D CNN significantly outperformed randomly initialized baselines when pretrained using their self-supervised objective, even when all training labels were included. Expanding on the jigsaw pretext task, Taleb et al. [125] demonstrated that including multiple MRI modalities in the pretraining phase was an improvement from single-modality pretraining and training from scratch. Since BraTS is multi-modal, it is unsurprising that representations from a single-modality pretrained network would trail multi-modality pretraining. They also applied generative methods to produce patches for underrepresented modalities. The patches used to construct the jigsaw puzzles were composed of segments from different modalities. In an effort to improve feature representations for boundary regions (and therefore downstream segmentation quality), Huang et al. [126] adopted a standard cuboid patch masking reconstructive task using a vision transformer, but applied a weighting factor to voxels belonging to regions where the intensity is rapidly changing. They also applied a symmetric position encoding that ensured equivalence of position encoding for corresponding left and right sides of the brain. An ablation study highlighted the merit of both of these improvements, evaluating on BraTS 2021. Unfortunately, it is difficult to compare the results highlighted by the aforementioned techniques because they were evaluated on different editions of BraTS.

A substantial number of studies have focused on using self-supervised pretraining for the detection of psychiatric diseases. Several studies have utilized the ADNI [127] and OASIS-3 [128] datasets to develop classifiers that can detect brain MRI scans of patients with Alzheimer’s disease (AD). Mahmood et al. developed 1D CNNs on time courses of resting state fMRI examinations to detect AD, schizophrenia, and autism [129]. They pretrained using a contrastive pretext task where the pairwise relationship consisted of a fragment of and the entirety of a time course, which improved AUC for all three classifiers. Evaluating on OASIS-3 [128], Fedorov et al. [130, 131] proposed contrastive pretraining where positive pairs consisted of paired fMRI and T1 MRI frames. The results are mixed, with fully supervised models outperforming pretrained models for T1 volumes and vice versa for fMRI volumes, for the task of AD detection. Leveraging the multiple examples per patient available in ADNI, Zhao et al. [132] suggested a pretext task that combines a basic autoencoder with mean squared error with a regularizer intended to enforce directionality in the latent space for representations of volumes from the same patient taken at two points in time. The regularizer maximizes the cosine between the difference between the representations of paired newer and older examples and a constant vector, $\mathbf {\tau }$. The idea is to learn representations such that adding a scalar multiple of $\mathbf {\tau }$ corresponds to an increase in brain age. Decoded MRI examples that varied along $\mathbf {\tau }$ indicated morphological differences associated with increased brain age. Lastly, pretrained models performed better than those initialized randomly. Expanding on this approach, these authors proposed a pretext task that clusters examinations with similar brain age, while still enforcing a direction of increasing brain age within neighbourhoods [133]. Their approach improved test AUC by 0.076 compared to their previous work. Dufumier et al. [134] report an improvement on AD detection in ADNI over full supervised learning, pretraining with Models Genesis [73], and SimCLR [23] when incorporating a weight into the standard contrastive objective corresponding to the difference in a continuous meta-variable, such as patient age. Other pretexts that have demonstrated improved performance in AD detection include contrastive learning with positive pairs composed from different orthogonal slice views and variable-length volumes [135], and positive pairs composed by pasting anatomically bounded components of one image onto another [136]. Moving the focus away from pathology, Osin et al. [137] were able to train a linear classifier using representations provided by a feature extractor pretrained to predict next-frame amygdala activity on fMRI. The classifier performed better than a CNN baseline at predicting demographic traits (e.g., age) and psychiatric traits according to clinical questionnaires (e.g., trait anxiety).

SSL has also proved useful for automatic white matter segmentation. In 2020, Lu et al. [138] devised a pretext task for white matter segmentation on diffusion MRI (dMRI) images from the openly available Human Connectome Project [139] that involved predicting density maps of white matter fiber streamlines. The labels for this pretext task were generated by applying a previously proposed tractography algorithm and producing a density map by aggregating the number of streamlines intersecting each voxel. After fine-tuning, the Dice score of the pretrained model was 0.137 greater than that of randomly initialized model. The following year, Lu et al. [140] extended this work by introducing a second pretext task that involved segmenting white matter based on labels computed using a registration-based algorithm available in a separate software package. They optimized the feature extractor on the first and then second pretext task (i.e., sequentially). Models pretrained using either or both of the pretext tasks outperformed the baseline. Interestingly, the authors did not compare sequential pretraining with simultaneous optimization of both objectives using separate decoder heads. Huang et al. [126] also applied their method (see previous paragraph) to the downstream task of white matter segmentation on the publicly available WMH dataset [141], but did not compare with a fully supervised baseline.

Studies have witnessed performance gains for other brain MRI tasks, such as brain anatomy segmentation [142–144], multiple sclerosis lesion segmentation [135, 143], and stroke lesion segmentation [143]. For instance, in an effort to improve brain anatomy segmentation, Chang et al. pretrained to solve two pretext tasks: (1) predicting the location of the vocal in the nearest supervoxel and (2) predicting the deformation field between the current volume and an atlas. Similar to [126], the first term promotes saliency in rapidly changing regions close to boundaries. The second term requires the encoder to produce features that highlight boundaries of larger structures, which are required for a registration task. Zoetmulder et al. [143] assessed the utility of supervised and self-supervised pretraining (with an auto-encoding pretext task) for multiple sclerosis lesion segementation, stroke lesion segmentation, and brain anatomy segmentation. They found that pretraining using MRI data resulted in better performance on downstream tasks than with natural images. They did not find that self-supervised pretraining was superior to supervised pretraining for all downstream tasks, but they employed a pretraining dataset that included classification and segmentation labels. While this is an important finding, the major utility of self-supervised learning is to leverage *unlabelled* data when labels are not available.

### Prostate MRI diagnostic tasks

The prostate segmentation task in MSD [101] is a benchmark for prostate semantic segmentation, where the task is to segment the peripheral zone and central gland of the prostate. Chaitanya et al. [145] applied a two-stage pretraining strategy, where an encoder is trained using standard contrastive learning in the first phase, and some decoder blocks are trained during the second phase to minimize a local contrastive loss that encourages dissimilarity among distinct patches in the same image. When fine-tuning, they appended the remainder of the decoder blocks, achieving greater Dice scores than randomly initializing the full model. Taleb et al. [125] (see Brain MRI diagnostic tasks section) also evaluated their approach using this dataset, but used a different test split.

The ProstateX benchmark dataset [146] contains segmentation maps for cancerous lesions of the prostate. Fernandez-Quilez et al. [147] observed that pretraining with SimCLR [23] improved downstream segmentation performance, compared random and ImageNet-pretrained initialization. They tailored the original stochastic transformation distribution such that it entailed plausible prostate MRI slices. Wang et al. [148] engineered a more complex pretext task for the same downstream task, which involved optimizing a contrastive learning objective where images from the same patient comprise a positive pair, and an augmentation classification objective. The two methods cannot be compared because they employed different evaluation protocols. Bolous et al. [149] also observed an improvement for the same downstream task with a private dataset when pretraining using a reconstructive pretext task.

### Cardiac MR segmentation

Segmentation of cardiac structures with machine learning is an extensively studied topic. Bai et al. [150] exploited the orientation of short-axis and long-axis planes as given in DICOM files to create a pretext task consisting of segmentation of fixed-size boxes placed at specific points along lines corresponding to bisection with other axes. The relative locations of the boxes is constant with respect to the major cardiac structures. Pretraining improved downstream segmentation of the left ventricle, right ventricle, and myocardium. Notably, optimizing the pretext and downstream objective simultaneously (in a semi-supervised fashion) yielded the greatest test Dice score. This study constitutes another successful example of leveraging domain knowledge available in unlabelled data. Ouyang et al. [151] demonstrated that self-supervised learning can replace standard training with labelled images for few-shot segmentation. They constructed superpixels from images and used randomly transformed copies of the original image for both support and query. Remarkably, self-supervised training resulted in better downstream segmentation of the left ventricle, right ventricle, and the myocardium on the Card-MRI dataset [152]. Other studies have integrated SSL into federated learning regimes [153] and meta-learning [154], citing improvement in performance for cardiac structure segmentation.

SSL has also proven useful for disease classification on cardiac MRI. Zhong et al. [155] found that, when corrupting cine cardiac MR volumes with random pixel shuffling, patch obfuscation, and entire frame dropout, reconstructive pretraining improved downstream classification of preserved versus reduced ejection fraction subtypes of heart failure. An ablation study demonstrated pretraining using each corruption, in isolation, also improved performance.

### Grading intervertebral disc degeneration

SSL pretraining has been applied successfully to a constellation of other tasks involving MRI data. One of the earliest studies employing SSL for MRI was conducted in 2017 by Jamaludin et al. [156], in which they pretrained a CNN on a spinal MRI dataset for the downstream task of grading disc degeneration disease according to the Pfirrmann system [157]. They pretrained to simultaneously solve two pretext tasks: (1) contrastive learning where positive pairs were longitudinal samples from the same patient and (2) classification of vetebral body level. The pretrained models consistently outperformed models trained from scratch, for varying levels of training label availability. Solving the same downstream task on a different private dataset, Kuang et al. [158] adopted a reconstructive pretext task, where inputs were distorted by applying different stochastic transformations to image regions corresponding to vertebral bodies, intervertebral discs, and the background. They used a previously described unsupervised segmentation algorithm to compute masks corresponding to these classes, avoiding the need for labels.

### Other MR diagnostic tasks

Studies have observed improved downstream performance when conducting self-supervised pretraining for other tasks with MRI data, including intracranial hemorrhage detection [159], anterior cruciate ligament tear detection [160], spinal tumour subtype classification [161], and abdominal organ segmentation [53, 105].

## Applications in ultrasound imaging

Evidence exists in support of pretraining machine learning models for diagnostic tasks with ultrasound (US) examinations. However, as outlined in Search methodology section, considerably fewer publications have explored self-supervised pretraining for US than for the preceding three types of radiological imaging. Although US examinations are typically represented as 3D tensors (4D when motion is displayed with colour), they are fundamentally different from CT and MRI in that the third dimension is temporal as opposed to spatial. However, like CT and MRI, there are occasions where a single image is sufficient to perform a particular diagnostic task.

### US breast malignancy detection

Nguyen et al. [53] explored the efficacy of BYOL [27] for the classification of breast US images from the public BreastMNIST dataset [54] as either normal, containing benign tumours, or containing malignant tumours. Although the paper is rife with terminological errors, it provides a benchmark for a nonconstrastive method on a public dataset. They found that pretraining with BYOL resulted in worse test performance than randomly initialized or ImageNet-pretrained weights. Perek et al. [162] arrived at a similar conclusion when trialling MoCo with a private dataset [24]. Proposing a video-specific pretext task instead, Lin et al. [163] pretrained an encoder-decoder architecture to restore a US video after randomly masking out entire frames and patches in the remaining frames. Upon performing semi-supervised fine-tuning for benign versus malignant lesion classification on a private dataset, masked video pretraining yielded $1\%$ greater accuracy compared to random initialization. Focusing instead on breast lesion semantic segmentation, Mishra et al. [25] pretrained an encoder-decoder to perform a deterministic edge detection or segmentation task that does not require machine learning. They performed experiments using two publicly available datasets (BUSI [164] & UDIAT [165]) and observed that SSL improved performance, with the gap increasing with less labelled training data availability. However, it is unclear which pretext task they selected for their downstream experiments.

### Echocardiography tasks

SSL has been cited as useful for a variety of echocardiography interpretation tasks. Anand et al. [166] sought to establish the performance of ubiquitous contemporary joint-embedding SSL methods for the task of view classification (e.g., SimCLR [23], MoCoV2 [167], BYOL [27], DINO [92]). Not only did they find that pretraining outperformed random and ImageNet-pretrained initialization, but they demonstrated that pretraining with more unlabelled data widened the performance gap. SimCLR and BYOL pretraining have been investigated for the task of left ventricle segmentation. Saeed et al. [168] observed that SimCLR pretraining generally resulted in the best Dice score, but the difference was small across label availability fractions. Surprisingly, BYOL pretraining generally resulted in worse performance than full supervision. The results appeared to be consistent across two public datasets: EchoNet-Dynamic dataset [169] and CAMUS [170]. To reduce redundancy of pretraining examples, they chose to use one randomly selected frame per clip during pretraining, despite using two labelled frames per clip for the downsteam task (one each for end-systole and end-diastole); it is possible that using more frames during pretraining may have improved performance. Dezaki et al. [171] devised a multifaceted pretext task customized for echocardiograms that consists of (1) reordering shuffled triplets of contiguous frames, (2) minimizing embeddings for contiuous frames and maximizing embeddings for temporally distance frames, and (3) minimizing the differences between embeddings of frames from multiple views corresponding to the same point in the cardiac cycle. Although fully supervised learning matched self-supervised pretraining when using all labels, SSL greatly improved performance when less labels were available. They observed similar results when evaluating on EchoNet-Dynamic.

### Assessment of thyroid nodules on US

US is often employed to assess thyroid nodules for possible malignancy. Zhao  & Yang [111] pretrained a classifier to distinguish between benign and malignant nodules, using the public TN-SCUI2020 [172] dataset. They integrated prior medical knowledge into their contrastive pretext task, which sought to minimize the differences between embeddings of handcrafted radiomics features and the original US image. Their method outperformed random initialization and pretraining with generic pretext tasks. Xiang et al. [173] also devised a custom pretext task for this problem, characterized by thyroid US modality classification. In addition to B-mode US, their downstream model received corresponding images from three US modalities, noting superior performance on their private dataset when pretraining as opposed to random or ImageNet-pretrained initialization. Guo et al. [174] focused on the related downstream task of grading nodules according to the widely adopted TI-RADS [175] system.

### Obstetric US tasks

Jiao et al. [176] described a custom US-specific pretext task consisting of predicting the order of 4 shuffled frames and predicting the continuous parameters of random affine transformations applied to the frames, which resulted in an improvement over training from scratch for the task of fetal plane detection. Chen et al. [117] (described in Section 6.1) observed a similar result for the same task. Focusing instead on segmenting the utero-placental interface, Qi et al. [177] pretrained a feature extractor for a customized jigsaw pretext task in which the permuted patches were sampled from image regions intersected the labelled region of interest. Results indicated marginal improvement with pretraining, but the custom pretext task did not outperform Jigsaw [22] for the majority of feature extractors studied. Of note is the fact that their pretext task cannot be considered SSL because, by definition, the pretext task is solved in the absence of labels.

### Other US diagnostic tasks

Liu et al. [178] pretrained an encoder-decoder model for the downstream task of classifying gastrointestinal stromal tumours from endoscopic US images, observing greater average performance than full supervision with ImageNet-pretrained initialization (albeit with greatly overlapping confidence intervals). Interestingly, they leveraged thyroid and breast US datasets for pretraining. Zhou et al. [179] found random permutation prediction to be a helpful pretext task for rheumatoid arthritis grading on US; however, the approach required manual region of interest labelling. Lastly, Basu et al. [180] proposed an US-specific contrastive pretext task that considered temporally separated frames from the same video as negative pairs, in addition to inter-video pairs. Positive pairs were frames separated temporally by no more than a predefined constant number of time steps. Further, they imposed a curriculum by gradually decreasing the minimum temporal distance constituting an intra-video negative pair. Intra-video negative pairs are important to consider because the anatomical context of an US video may differ dramatically throughout its duration. However, the authors did not address how negative pair sampling would be considered for cases where the probe is kept stationary throughout the video. The authors evaluated their approach on a private dataset for gallbladder malignancy detection and on the public POCOVID-Net [181] lung US dataset for COVID-19 classification, citing performance superior to ImageNet-pretrained initialization, SimCLR [23], and MoCoV2 [167].

## Assessment & future directions

### Evidence for SSL pretraining

#### Comparison to random initialization

The previous sections of this work illustrate the usefulness of self-supervised pretraining in deep learning for diagnostic tasks with radiological images. For each of the four major modalities investigated, there are multiple studies that report an improvement in downstream performance metrics when initializing feature extractors with SSL-pretrained weights, generally compared to random weight initialization in the fully supervised setting.

In most cases, studies demonstrated that pretraining was useful either as a first step using all labelled data, or that pretraining was particularly helpful in low label availability settings. When labels are completely available for a downstream task, there is a wide variation in the change in performance on test data. Some studies report marginal to no improvement [41, 177, 178], while others report significant gains [133, 138, 158, 160]. Naturally, there are myriad reasons for such variability, including dissimilar pretext tasks, evaluation protocol differences, modality-specific noise, dataset volume and diversity, and downstream task difficulty.

The results of this review overwhelmingly suggest that pretraining with self-supervised learning is likely to result in improved performance on downstream supervised learning tasks, compared to randomly initialized supervised learners. Practitioners should consider trialling pretrained feature extractors during model development.

#### The power of ImageNet-pretrained weights

The vast majority of the methods explored in this review compared their pretrained models to the fully supervised setting where weights are randomly initialized. Many also compared the results of their custom SSL method to previously proposed SSL methods that are not geared toward any specific imaging distribution. However, a fraction of studies compared their pretrained feature extractors to the ubiquitously employed ImageNet-pretrained weights. It is reasonable to compare against ImageNet-pretrained weights because several medical computer vision models are initialized with ImageNet-pretrained weights [43]. Indeed, many studies reported that ImageNet-pretrained weights fared better than random initialization, making them a stronger baseline against which to compare. Crucially, multiple studies reported cases where 2D CNNs or vision transformers did not appreciably outperform ImageNet-pretrained initialization [41, 42, 53, 73–75]. We therefore advise authors of future SSL studies to compare their approaches to fully supervised baselines with random initialization *and* ImageNet-pretrained initialization where applicable.

A frequently absent experimental setting is the assessment of the effect of initializing feature extractors with ImageNet-pretrained weights *prior* to self-supervised pretraining. The small set of studies that performed this comparison observed that the best performance in downstream radiological imaging interpretation tasks was achieved by setting the initial weights of the feature extractors to ImageNet-pretrained weights [39, 43]. Future studies should include this experiment in their evaluation protocol.

Of course, it is necessary to acknowledge that publicly available ImageNet-pretrained weights do not exist for all feature extractor architectures (e.g., 3D CNNs), and that fully supervised pretraining can be prohibitively expensive.

#### Utility of SSL in low-label settings

Aside from direct comparisons to fully supervised counterparts using the same dataset, several studies have established the benefit of self-supervised pretraining in scenarios where labels are not provided for all available examples. Typically, such claims are established by comparing performance of fully and self-supervised models at different fractions of label availability, limiting the amount of data available for supervised fine-tuning on a downstream task [43, 99, 106, 117, 124, 171]. Some studies reported changes in downstream performance when larger unlabelled datasets that dwarfed the available labelled examples were leveraged for pretraining [39, 100, 166]. Some studies even demonstrated that pretraining with unlabelled data geared for a different downstream task but that was collected using the same modality can improve downstream performance [90, 126, 134, 178]. In the extreme scenario of few-shot learning, self-supervised objectives may be employed during training [151]. The considerable amount of evidence outlined in this review suggests that practitioners should leverage unlabelled data when available and pretrain feature extractors using SSL.

#### Relative dearth of ultrasound research

As depicted in Fig. 2a, the number of papers eligible for inclusion in this review concerning US data is less than half of the number included for X-ray, CT, or MRI. Hence, there exists a need for (1) more investigations that quantify the impact of preexisting SSL pretraining tasks for US tasks and (2) studies that modify preexisting or propose novel SSL methods that are suited to the US modality. US presents additional challenges for machine learning systems compared to the other modalities, such as increased noise, the temporal dimension, acquisition-related differences in probe movement and orientation, motion artefacts, and geometrical differences across probe types and manufacturers. As a result, further work is warranted in determining the types and aspects of pretext tasks suitable for US.

### Theoretical support for empirically validated methods

The majority of the studies presented in this review provide SSL methods that are presented as task-specific, instead of applying preexisting methods to ne. Some deviate wildly from previous work [77, 136, 142, 143], and others are incremental changes to previously explored pretext tasks [115, 121]. The pretext tasks put forth in such studies are often fashioned with clinical and/or background knowledge about the downstream task, but are mostly justified by intuition. The arguments for further use of the proposed methods typically consists entirely of empirical validation. Multiple such studies boast superior performance of their methods boast empirical results but do not establish statistically significant improvements [85, 178, 182].

As discussed in Theoretical support section, some SSL methods have received theoretical justification in terms of performance on downstream task. Many studies discussed in this survey employ such justified methods, such as SimCLR [23] and Barlow Twins [26]. These methods are guaranteed to improve performance on downstream tasks as long as the labels for positive pairs would be the same in the downstream task. For example, Fernandez-Quilez et al. [147] employed SimCLR with a modified transformation distribution that captured differences between positive pairs that would not constitute a change of label. Azizi et al. [43] also employed SimCLR, but expanded the pairwise relationship to include multiple acquired views of the same pathology. Applying custom data augmentation transformations that do not change the label distribution in the downstream task or defining the pairwise relationship based on preexisting clinical knowledge are viable strategies for the successful application of theoretically justified joint-embedding SSL methods. Such clinical knowledge may come “for free" in that it does not require further labelling — practitioners could consider sources such as multiview examinations, multimodal studies, accompanying radiology reports, and DICOM tags. Future methods should strive to apply theoretically justified approaches to SSL pretraining where possible; otherwise, statistical significance testing should be conducted when claims are made regarding the superiority of novel methods.

### Comparable and reproducible benchmarks

A longstanding problem in machine learning for medical imaging is the lack of public datasets, which thwarts replicability of results. A considerable number of studies in this review presenting novel SSL methods for radiological imaging tasks conducted their evaluations on private datasets only. As a result, many of the results presented are not directly commparable. This review was only able to directly compare studies for a limited set of downstream tasks where authors reported performance on public datasets. Authors suggesting novel SSL methods are encouraged to evaluate their methods using public datasets, or to include results on public datasets in addition to their private datasets (e.g., [148, 171]). When evaluating on public datasets, researchers should use train/test splits that are identical to preceding studies. Furthermore, authors should endeavour to utilize identical pretraining and training sets when evaluating their approach on standard public datasets. To promote usage of public benchmarks in future studies, Tables 6, 7, 8, and 9 detail all public datasets referenced in this review, providing URLs for access.

**Table 6:** The public X-ray datasets referenced in this review, including links to request or download the data

Name [Citation]	Description	Examples	Patients
CheXpert [6]	A fully manually annotated 14-class dataset of chest X-rays.	${224\,316}$	${65\,240}$
ChestX-ray14 [33]	A 14-class dataset of chest X-rays with labels extracted from radiology reports.	${112\,120}$	${30\,805}$
ChestMNIST [34]	Identical to ChestX-ray14. Part of MedMNIST [54].	${112\,120}$	${30\,805}$
COVIDx CXR-2 [56]	chest X-rays labelled for the presence or absence of COVID-19.	${19\,203}$	${16\,656}$
MIMIC-CXR [65]	Chest X-rays, metadata, and free text reports. Same label categories as CheXpert. Some labels were manually determined, and others were automatically assigned using the reports.	${371\,920}$	${65\,079}$
RSNA Pneumonia [183]	Chest X-rays with bounding box labels for bacterial and viral pneumonias	${30\,000}$	${12\,274}$
PneumoniaMNIST [184]	Paediatric chest X-rays labelled for the presence or absence of pneumonia. Part of MedMNIST.	5856	5856

**Table 7:** The public CT datasets referenced in this review, including links to request or download the data

Name [Citation]	Description	Images	Exams	Patients
LIDC-IDRI [71]	Chest CT exams labelled for lung nodule classification and segmentation.	-	1018	1010
LUNA2016 [72]	Chest CT exams labelled for the presence of lung nodules.			
COVID-CT [89]	Chest CT exams labelled for the presence or absence of COVID-19.	812	-	271
NIH Pancreas-CT [95]	Abdominal contrast-enhanced CT scans with pancreas segmentation labels	-	82	80
MSD Pancreas [101]	Abdominal CT exams with segmentation labels for pancreas parenchyma, cysts, and tumours. Part of the Medical Segmentation Decathlon [185].	-	420	-
BTCV [103]	Abdominal CT exams with segmentation labels for 13 organs.	-	50	-
KiTS19 [110]	CT exams labelled for kidney tumour segmentation	-	300	300
WHS-CT [119]	Axial CT exams with segmentation labels for the ventricles and atria of the heart.	-	60	60
RSNA-PE [186]	Chest CT exams annotated with instances of pulmonary emboli.	${2\,995\,147}$	${12\,195}$	${12\,195}$

**Table 8:** The public MRI datasets referenced in this review, including links to request or download the data

Name [Citation]	Description	Exams	Patients
BraTS [123]	MRI exams labelled for brain tumour segmentation and classification. The benchmark has been updated and previous versions are available.	8000	2000
ADNI [127]	Brain MRI exams with labels for normal controls, mild cognitive impairment, and Alzheimer’s disease	2641	811
OASIS [128]	Brain MRI exams with segmentation labels, patient characteristics, and labels for Alzheimer’s disease	2842	1379
HCP [139]	Unannotated multi-modal MR scans	-	1206
WMH [141]	Brain MRI exams with labels for white matter hyperintensities 150	150	
ProstateX [146]	Prostate MRI studies labelled for localization and classification of prostate lesions	538	344
Card-MRI [152]	Cardiac MRI exams with labels for ventricular blood volume and myocardium segmentation	60	60

**Table 9:** The public US datasets referenced in this review, including links to request or download the data

Name [Citation]	Description	Images	Videos	Patients
EchoNet-Dynamic [169]	Echocardiography videos with end diastolic and end systolic volume labels	-	${10\,030}$	${10\,030}$
TN-SCUI2020 [172]	Thyroid US videos with segmentation labels for thyroid nodules	-	3644	3644
POCOVID-Net [181]	Links to lung US videos labelled for COVID-19, other viral pneumonia, bacterial pneumonia, and healthy lung.	1103	64	-
CAMUS [170]	Echocardiograms labelled for segmentation and volume estimation.	-	500	500
BUSI [164]	Breast US images with classification labels for normal, benign lesion, and malignant lesions.	780	-	600
BreastMNIST [164]	Identical to BUSI [164]. Part of MedMNIST.	780	-	600
UDIAT [165]	Breast US images labelled as benign or malignant.	163	-	163

### The impact of pretraining on generalizability

Machine learning models trained for tasks involving radiological images are utterly susceptible to performance drops under distributional shift [8]. Biases can be introduced by the distribution of confounding or mediating variables in the training set, such as labelling discrepancies, patient demographics, acquisition technique, and device manufacturer. External validation is therefore a pivotal pre-deployment step. Some studies in this review reported improvement in performance on external test sets when self-supervised pretraining was conducted [43, 64, 179], but further work is required to confidently characterize this phenomenon.

## Limitations

Despite the comprehensiveness of this survey, some limitations must be noted. First, the study’s exclusive focus on self-supervision excluded adjacent categories of machine learning used in practice to produce feature extractors for transfer learning. Supervised pretraining on datasets of medical images can also produce feature extractors [187]. However, it presupposes access to sufficiently large quantities of labelled data – an uncommon situation in medical imaging. Semi-supervised learning is another family of methods designed for settings where unlabelled examples outnumber labelled examples. In contrast to SSL, semi-supervised methods involve the simultaneous optimization of an unsupervised objective and a supervised objective with task-specific labels. Generally, the pretraining phase of SSL produces a generic feature extractor without knowledge of the downstream task; whereas, semi-supervised training is directly linked to the task of interest. Since semi-supervised learning is not within the scope of this survey, we refer readers to other works covering its applications in medical imaging [188–190].

Although this survey’s scope was limited to applications in X-ray, CT, MRI, and ultrasound, it is important to acknowledge that SSL methods have been applied in other areas of medical imaging, such as histopathological, dermatologic, and endoscopic images [11, 12]. Practitioners working with modalities less represented in the literature may benefit by examining techniques described for different modalities, as some methods may be broadly applicable.

## Conclusions

This work reviewed a range of recent studies across modalities, datasets, and methods that explored the impact of self-supervised pretraining for the automation of diagnostic tasks in radiological imaging. The consensus observed in the majority of the publications included in this survey suggest that SSL pretraining using unlabelled datasets generally improves the performance of supervised deep learning models for downstream tasks in radiography, computed tomography, magnetic resonance imaging, and ultrasound. The findings substantiate the utility of unlabelled data in radiological imaging, thereby reducing the prohibitive expense of expert labelling. Practitioners should therefore consider self-supervised pretraining when unlabelled data is abundant. Future work in SSL for radiological imaging should focus on developing and/or applying theoretically justified methods that capitalize on clinical knowledge, further exploring SSL for problems in ultrasound, and ascertain the effect of SSL on generalizability.

### Supplementary Information

**Supplementary Material 1.**

## Data Availability

Data sharing is not applicable to this article as no datasets were generated or analysed during the current study. Search queries for studies included in this article are available in Appendix A. Links to public datasets referenced in this article are included in Appendix B. Spreadsheets containing results reported for commonly used benchmark datasets can be found in the Supplementary Dataset.

## Abbreviations

AUC: Area under the receiver operating characteristic curve
CNN: Convolutional neural network
CT: Computed tomography
CXR: Chest X-ray
MRI: Magnetic resonance imaging
SSL: Self-supervised learning
US: Ultrasound
