# Supplementary material for: A survey of the impact of self-supervised pretraining for diagnostic tasks in medical X-ray, CT, MRI, and ultrasound
Source: BMC Med Imaging. 2024 Apr 6;24:79. doi: 10.1186/s12880-024-01253-0 (PMC10998380; doi:10.1186/s12880-024-01253-0)
Supplement: Supplementary file 1 — Supplementary Material 1. [file 12880_2024_1253_MOESM1_ESM.zip › Appendix.pdf]

Appendix A: Database Queries

Four databases were queried in November 2022 to gather the set of candidate studies for review, including Scopus[1], IEEE Xplore Digital Library[2], ACM Digital Library[3], and PubMed[4]. Below are the queries, along with the number of results returned by each database.

Scopus (856 results)

```
(
  TITLE-ABS-KEY ( ( "self-supervis*" OR
                      "{contrastive learn*}" ) )
  AND
  TITLE-ABS-KEY ( ( "{*medical imag*}" OR
                     "X-ray*" OR
                     ultrasound* OR
                     "{computed tomography}" OR
                     "CT" OR
                     "{magnetic resonance imaging}" OR
                     mri ) )
)
```

IEEE Xplore Library (328 results)

```
((("All Metadata":"self-supervis*" OR
   "All Metadata":"contrastive learn*")
  AND
  ("All Metadata":"*medical imag*" OR
   "All Metadata":"X-ray" OR
   "All Metadata":"computed tomography" OR
   "All Metadata":CT OR
   "All Metadata":"ultrasound" OR
   "All Metadata":"magnetic resonance imaging" OR
   "All Metadata":MRI))
  OR
  ("Index Terms":"self-supervis*"
   AND
   ("Index Terms":"*medical imag*" OR
   "Index Terms":"X-ray*" OR
   "Index Terms":"computed tomography" OR
   "Index Terms":CT OR
   "Index Terms":"ultrasound" OR
   "Index Terms":"magnetic resonance imaging" OR
   "Index Terms":MRI))
)
```

ACM Digital Library (8 results)

```
(
  Title:("medical image" OR "medical images" OR "medical imaging"
         OR "biomedical image" OR "biomedical images" OR
         "biomedical imaging" OR xray OR "computed tomography" OR
         ct OR ultrasound OR "magnetic resonance imaging" OR mri)
  OR
  Abstract:("medical image" OR "medical images"
            OR "medical imaging" OR "biomedical image" OR
```

[1] https://www.scopus.com

[2] https://ieeexplore.ieee.org

[3] https://dl.acm.org

[4] https://pubmed.ncbi.nlm.nih.gov

```
                "biomedical images" OR "biomedical imaging" OR xray OR
                "computed tomography" OR ct OR ultrasound OR
                "magnetic resonance imaging" OR mri)
  OR
  Keywords:("medical image" OR "medical images" OR
                "medical imaging" OR "biomedical image" OR
                "biomedical images" OR "biomedical imaging" OR xray OR
                "computed tomography" OR ct OR ultrasound OR
                "magnetic resonance imaging" OR mri)
)
AND
(
  Title:"selfsupervised" OR Abstract:"selfsupervised" OR
  Keywords:"selfsupervised" OR Title:"selfsupervision" OR
  Abstract:"selfsupervision" OR Keyword:"selfsupervision"
)
```

PubMed (42 results)

```
(
  self-supervis*[tiab] OR "self supervis*"[tiab] OR
  "contrastive learn*"[tiab]
)
AND
(
  Machine Learning[Mesh:NoExp] OR deep learning[mesh:noex]
)
AND
(
  "medical imag*"[tiab] OR diagnostic imaging[mesh] OR mri[tiab] OR
  ct[tiab] OR "computed tomography"[tiab] OR "x-ray"[tiab] OR
  ultrasound[tiab]
)
```

Appendix B: Public Datasets

Several studies reviewed in this work utilized public datasets. To promote replicability via the usage of public benchmarks, we provide brief descriptions and links for these datasets. Where available, we also provide the number of examples in the dataset and the number of patients whose studies are included. Tables 6, 7, 8, and 9 list the public datasets for X-ray, US, CT, and MRI respectively.

The Supplementary Dataset contains results reported by studies on common public benchmark datasets. It consolidates the contents of Tables 1, 2, 3, 4, and 5.
